# Donor age and body weight determine the effects of fecal microbiota transplantation on growth performance, and fecal microbiota development in recipient pigs

**DOI:** 10.1186/s40104-022-00696-1

**Published:** 2022-04-11

**Authors:** Xiaofan Wang, Tsungcheng Tsai, Bin Zuo, Xiaoyuan Wei, Feilong Deng, Ying Li, Charles V. Maxwell, Hua Yang, Yingping Xiao, Jiangchao Zhao

**Affiliations:** 1 Department of Animal Science, Division of Agriculture, University of Arkansas, Fayetteville, AR USA; 2 Guangdong Provincial Key Laboratory of Animal Molecular Design and Precise Breeding, College of Life Science and Engineering, Foshan University, Foshan, 528225 China; 3 State Key Laboratory for Managing Biotic and Chemical Threats to the Quality and Safety of Agro-products, Institute of Agro-product Safety and Nutrition, Zhejiang Academy of Agricultural Sciences, Hangzhou, China

**Keywords:** Colonization, FMT, Growth performance, Swine gut microbiome

## Abstract

**Background:**

The application of fecal microbiota transplantation (FMT) to improve swine growth performance has been sporadically studied. Most of these studies used a single microbiota source and thus the effect of donor characteristics on recipient pigs’ fecal microbiota development and growth performance is largely unknown.

**Results:**

In this study, we collected feces from six donors with heavy (H) or light (L) body weight and different ages (d 42, nursery; d 96, growing; and d 170, finisher) to evaluate their effects on the growth performance and fecal microbiota development of recipient pigs. Generally, recipients that received two doses of FMT from nursery and finisher stages donor at weaning (21 ± 2 days of age) inherited the donor’s growth pattern, while the pigs gavaged with grower stage material exerted a numerically greater weight gain than the control pigs regardless of donor BW. FMT from heavier donors (NH, GH, and FH) led to the recipients to have numerically increased growth compared to their lighter counterparts (NL, GL, and FL, respectively) throughout the growing and most finishing stages. This benefit could be attributed to the enrichment of ASV25 *Faecalibacterium*, ASV61 *Faecalibacterium*, ASV438 *Coriobacteriaceae_*unclassified, ASV144 *Bulleidia*, and ASV129 *Oribacterium* and decrease of ASV13 *Escherichia* during nursery stage. Fecal microbiota transplantation from growing and finishing donors influenced the microbial community significantly in recipient pigs during the nursery stage. FMT of older donors’ gut microbiota expedited recipients’ microbiota maturity on d 35 and 49, indicated by increased estimated microbiota ages. The age-associated bacterial taxa included ASV206 *Ruminococcaceae*, ASV211 *Butyrivibrio*, ASV416 *Bacteroides*, ASV2 *Streptococcus*, and ASV291 *Veillonellaceae*. The body weight differences between GL and GH pigs on d 104 were associated with the increased synthesis of the essential amino acid, lysine and methionine, mixed acid fermentation, expedited glycolysis, and sucrose/galactose degradation.

**Conclusions:**

Overall, our study provided insights into how donor age and body weight affect FMT outcomes regarding growth performance, microbiota community shifts, and lower GI tract metabolic potentials. This study also provided guidance to select qualified donors for future fecal microbiota transplantation.

**Supplementary Information:**

The online version contains supplementary material available at 10.1186/s40104-022-00696-1.

## Introduction

The gut microbial ecosystem, possessing multidimensional biological functions, has become a crucial factor affecting various phenotypic characteristics of the host [1–6]. Fecal microbial transplantation (FMT), a microbial intervention method by transplanting fecal microbiotas from healthy donors to diseased recipients, has been practiced for treating a variety of gut diseases, such as recurrent *Clostridium difficile* infections, inflammatory bowel disease, chronic constipation, and irritable bowel syndrome since the mid-twentieth century [7–9]. Also, FMT was identified as one of the most promising next-generation therapeutic approaches for cardiometabolic, autoimmune, obesity, and mental disease remedies [10–12].

The pig is an ideal model for studying human infectious diseases due to possessing similar human organ structures [13]. FMT studies in pigs have broadened our understanding of the mechanisms leading to improved gut community function [14]. Fecal feedback technique has been used with farm animals to conquer enteric infections [15–17]. Works from Kansas State University elicited that FMT can enhance immune tolerance and inhibit respiratory infections in swine [18, 19]. In addition, a study using a gavage-fed fecal microbiota suspension from an adult Jinhua pig into different newborn species (Duroc × Landrace × Yorkshire) increased growth performance, reduced diarrhea incidence, and enhanced the intestinal barrier function and immune system of the recipients [20]. Similarly, our previous study introduced weaning piglets with growth-stage donor microbiota which improved their average daily gain [21]. Furthermore, FMT from Jinhua pigs with “obese” characteristics to antibiotic-treated mice increased adiposity in the recipients [22]. However, deleterious effect of FMT was also reported that transplanting fecal microbiota from high feed efficiency pigs to pregnant sows reduced the growth rate in offspring [23].

Many attempts have been made to evaluate FMT as a tool to improve swine growth performance [20, 21, 24]. The donor characteristics that exert the maximum beneficial effects and duration on the recipients are still unknown. Identifying a quality donor is key to optimizing FMT efficacy and potential therapeutic and production practices [22, 25, 26]. In addition, the ecological dynamics of the microbiome community affected by FMT are still elusive, and many related questions remain unanswered. For example, how does an exogenous microbiota change the recipient’s gut microbial structure? Are the colonization efficiencies in the recipients associated with donor age and phenotype? In this study, we hypothesized that differences in donor age and growth performance affects FMT outcomes in recipient piglets. To test our hypothesis, we gave weaning piglets a fecal microbiota suspension from donor pigs of different ages and body weights. The recipient phenotypes and fecal microbiota developments, up to market weight, were closely tracked to study the community biological and ecological changes.

## Materials and methods

### Ethics statement

The animals were managed according to the Institutional Animal Care and Use Committee of the University of Arkansas (approved under IACUC #19024).

### Animal management and study design

#### Donor pigs and processing of fecal material

Fresh fecal samples were collected from six donors: a high and low (within the top and bottom 10% of the overall ranking from approximately 336 pigs) body weight (BW) of the nursery (42 days old), growing (96 days old), and finishing (170 days old) stage pigs, respectively. All donor pigs were raised free of feed antibiotics and pharmaceutical levels of zinc and copper prior to fecal collection. Fecal samples (2 g each) were suspended in 18 mL of sterile PBS with 20% glycerol in a sterile Whirl-Pak filter bag with 0.33-mm pores and then subjected to a 2-min high speed homogenization by a Stomacher™ 400 (Seward Ltd., West Sussex, UK). The filtered microbial suspension was then transferred into 50-mL conical tubes and stored at − 80 °C [21]. Before use, serial dilutions of each suspension were plated on brain heart infusion agar and incubated at 37 °C for 48 h to determine CFU/mL. Based on the CFU/mL, mixtures were normalized to 2.4 × 10^8^ CFU/mL using the same glycerol/PBS solution batch.

#### Animals and study design

A total of 80 weaned piglets (PIC1050 × DNA600; 21 ± 2 days of age) with an average BW of 6.5 kg were transferred to an onsite nursery facility at the University of Arkansas Swine Research Unit. On weaning day, piglets were sorted by gender and were then gavaged with a dose drenching syringe (Cotran Corporation, Portsmouth, RI, U.S.A.) for two consecutive days with 10 mL of one of the following seven pre-thawed transplant solutions: PBS contained 20% glycerol v/v (Con), fecal suspension from heavy or light BW of the nursery (NH and NL), grower (GH and GL), and finisher (FH and FL) stages. Three replicate pens, each with two male and two female pigs were recruited to each treatment (*n* = 12) except FL groups, which contained two replicate pens (*n* = 8). At the end of the nursery stage, pigs were transferred to a grower/finisher facility to study the longitudinal response of FMT until market weight.

All pigs were fed the same 7-phase dietary regimens with a 14-day duration per phase (phase 1: d 21–35; phase 2: d 35–49; phase 3: d 49–63; Additional file 1: Table S1) in the nursery stage. The length of each phase in the grower (G 1.1: d 63–76; G 1.2: d 76–90; G 2.1: d 90–104; G 2.2: d 104–118) and finisher stages (F 1.1: d 118–127; F 1.2: d 127–145; F 2.1: d 145–159; F 2.2: d 159–174) was determined as the average BW of pigs reached target BW based on nutrient levels formulated for a given phase (Table S2). All pigs went to market on d 174. All diets met or exceeded the nutrient requirements (NRC, 2012) [27] for pigs at each growth stage and were devoid of antibiotics and pharmaceutical levels of Zn and copper. Pigs were housed in 1.50m × 1.20 m and 1.5m × 3.0 m total slatted pens with ad libitum access to feed and water during nursery and growing/finishing phases, respectively.

### Growth data recording and sample collection

Individual BW was recorded on the weaning day prior to FMT and again at the end of each phase change during the nursery period (d 35, d 49, and d 63), while BW was collected at middle and the end of each phase during the grower (G 1: d 76 and d 90; G 2: d 104 and d 118) and finisher (F 1:d 127 and d 145; F 2; d 159 and d 174) periods. Two pigs were randomly selected from each pen (*n* = 40) for fecal swab sampling (Puritan Opti-Swab, PuritanMedical Products, Guilford, ME, USA) at weaning, and the same pigs were sampled repeatedly at each phase change throughout the entire trial. Additional swab samples were collected on d 25 and 32 to trace the short-term effects of the FMT. All swabs (40 swabs/time points and eight different time points) were stored at − 80 °C until further processing.

### DNA extraction and 16S rRNA gene amplicon sequencing

A total of 100 μL of fecal suspension (from each swab sample or donor microbiota solutions, *n* = 326) was subjected to bacterial genomic DNA extraction using the PowerLyzer PowerSoil DNA Isolation Kit (Qiagen, Germantown, MD, USA) by following the manufacturer’s protocol. Library construction for 16S rRNA amplicon sequencing followed the strategy that was described previously [21]. Extracted DNA was validated for quality and quantity by a NanoDrop One C (Thermo Fisher Scientific, Wilmington, DE, USA) and amplified by polymerase chain reaction (PCR) targeting the bacterial 16S rDNA V4 region. All DNA templates were diluted to 10 μL/mL before adding to a PCR plate. Accuprime Pfx Supermix (Invitrogen, Carlsbad, CA, USA) was selected as the PCR reagent to guarantee high fidelity. The primers used for the 16S rDNA V4 region were F: 5′– GTGCCAGCMGCCGCGGTAA − 3′ and R: 5′– GGACTACHVGGGTWTCTAAT − 3′ with a dual-index sequencing strategy that was developed by Kozich et al. [28] on an Illumina Miseq platform. Negative control and Mock communities (ZymoBIOMICS™ Microbial Community Standard; Zymo, Irvine, CA, USA) were included in the PCR for quality control. Individual PCR products were normalized by a SequalPrep Normalization Plate Kit (Invitrogen, Carlsbad, CA, USA) to obtain equivalent DNA densities between samples. Normalized amplicons were validated by a Qubit 3 Fluorometer (Thermo Fisher Scientific, Waltham, MA), followed by pooling and homogenization. The final pool underwent another cycle of quality and quantity evaluation by using an Agilent Bioanalyzer 2100 (Agilent, Santa Clara, CA, USA) and quantitative RT-PCR, respectively, before Miseq sequencing. NaOH solution (0.2 mol/L)-denatured final pool of DNA library and Phix were diluted by HT1 buffer at a 4:1 ratio before loading onto an Illumina MiSeq Reagent Kit v2 (500 cycles) cartridge.

### Sequence processing and bioinformatics analysis

Quality control (QC)-passed FASTQ files generated by the Illumina Miseq were imported into QIIME2 (2020.02 release). The demultiplexed sequences were processed using Deblur integrated with QIIME2 with default parameters including paired reads joining, length trimming, quality filtering, denoising (Deblur), classification (Greengenes reference database;13–8 version; 99% similarity), and sequence clustering. Chimeric sequences and singletons were removed by the Deblur program. Amplicon sequence variants (ASVs) were clustered based on 100% identity. All samples were rarefied to the minimum sample depth at 5907 reads to reduce the effects of sequencing depth on alpha (Shannon index, observed ASVs) and beta (Bray-Curtis) diversity measures.

Permutational multivariate analysis of variance (PERMANOVA) and analysis of similarity (ANOSIM) were conducted in QIIME2. Differentially represented bacterial members between groups were determined using Galaxy LEfSe (https://huttenhower.sph.harvard.edu/galaxy/). A feature table with metadata was imported into R-studio for further analysis and visualization. Spearman correlation was used to identify BW-associated bacterial features (R > 0.25). Regression-based Random Forest and the predict function in the R platform were used for estimated age (EMA) evaluation. SourceTracer software was used to calculate the contributions of the source (e.g., donor microbiota) to the sink microbiota (e.g., recipient pigs). Phylogenetic Investigation of Communities by Reconstruction of Unobserved States 2 (PICRUSt2) was performed to study the predictive metabolic potentials of fecal microbiota [29]. KEGG pathway abundances were calculated based on gene family mapping. Statistical Analysis of Metagenomic Profiles (STAMP) software package (v2.1.3) was used to further analyze the significances (two-sided White’s non–parametric *t*-test; *P* < 0.05) of altered KEGG pathways and data visualization [30].

### Statistical analysis of growth performance and feed efficiency

Growth performance measures, including BW and ADG, were analyzed by GLM procedure of SAS 9.3 (SAS Institute, Inc., Cary, NC, USA) as a randomized complete design. Treatment was used as a fixed effect, and the individual pig was used as an experimental unit in the analysis of variance. Statistically significant was set at α ≤ 0.05.

## Results

### Donor growth stage and performance determine the effect of FMT on the growth performance of recipients

Donor growth performance showed a pattern of influence that FMT had on recipient growth rate and final body weight. Although not statistically significant with small sample size, FMT from heavier donors (NH, GH, and FH) led to the recipients to have numerically increased growth compared to their lighter counterparts (NL, GL, and FL, respectively) throughout the growing and most finishing stages (Table 1). In addition, pigs that received FMT from heavier donors had greater body weight numerically than the control group at most of the time points (e.g. d 63, Con, NH, GH and FH = 22.0 ± 0.97, 23.6 ± 0.97, 23.9 ± 0.97, and 23.6 ± 0.93 kg, respectively, *P* = 0.72; d 174, Con, NH, GH and FH = 132.0 ± 4.28, 137.6 ± 4.29, 136.9 ± 4.48, and 134.6 ± 4.09 kg; *P* = 0.22), regardless of the donors’ growth stage. On the contrary, recipient pigs from the lighter donors of the nursery and finishing stages (NL and FL) had lighter body weight than the control group numerically starting from the end of growing phase 1.2 to the end of the finishing stage (e.g. d 104, Con, NL, and FL = 56.6 ± 2.87, 50.8 ± 2.73, and 50.8 ± 3.41 kg respectively; *P* = 0.08). Interestingly, the effect of the donor phenotype is also stage-dependent. Pigs that received FMT from the GL (i.e., growth stage with lighter body weight) donors had the best performance among those that received FMT from lighter donors (i.e., NL, GL, and FL). Although these pigs were lighter than those in the control group from the end of growing phase 1.2 to finishing phase 1.1, they improved by the end of finishing phase 1.2 and concluded as the heaviest pigs at the completion of the experiment (137.8 ± 4.29 kg) with a 5.8 kg heavier BW than the control group (132.0 ± 4.29 kg). On market day, recipients that received NH, GH, and FH microbiota transplantation were 5.6, 6.9, and 2.6 kg heavier than the control group, respectively. However, pigs that received FMT from the NL (126.6 ± 4.09 kg) and FL (124.4 ± 5.11 kg) groups were 5.4 and 7.6 kg lighter than the control group, respectively (Table 1). In addition, average daily gain (ADG) of NH and FH pigs were numerically higher than NL (0.857 ± 0.028 vs. 0.782 ± 0.028 kg) and FL (0.837 ± 0.027 vs. 0.771 ± 0.030 kg) pigs throughout the experimental period, respectively (Table 1). Generally, growing stage donor microbiota provided the greatest promoting effect benefit to growth rate compared to nursery and finishing donors regardless donor BW, yet caution should be taken when selecting donors from nursery and finisher stages.

**Table 1:** Effects of fecal microbial transplantation from donors with different growth stage and performance on the body weight and average daily gain of recipient pigs (LS means)

	Control	NL	NH	GL	GH	FL	FH	SEM	*P*-value
Body weight, kg									
End of NP 1; d 35	7.4	7.8	7.7	7.8	8.2	7.9	8.0	0.3	0.61
End of NP 2; d 49	12.7	12.4	14.1	13.3	13.5	13.6	13.3	0.6	0.34
End of NP 3; d 63	22.0	22.4	23.6	23.8	23.9	22.9	23.6	1.0	0.72
End of G 1.1: d 76	29.8	29.6	32.9	32.9	33.9	31.8	32.1	1.4	0.19
End of G 1.2: d 90	42.1	39.1	43.6	41.7	47.1	41.5	43.4	2.0	0.19
End of G 2.1; d 104	56.6	50.8	59.4	56.2	62.5	50.8	56.7	2.9	0.08
End of G 2.2: d 118	74.4	66.3	75.0	72.7	75.5	64.1	72.8	3.7	0.25
End of F 1.1: d 127	84.4	77.1	86.5	83.6	86.6	73.5	82.9	4.1	0.28
End of F 1.2: d 145	105.4	98.9	108.1	106.8	108.0	94.1	105.7	4.5	0.31
End of F 2.1: d 159	117.9	111.8	119.3	121.2	122.3	107.6	117.9	4.4	0.30
End of F 2.2; d 174	132.0	126.6	137.6	137.8	136.9	124.4	134.6	4.4	0.22
Average daily gain, kg									
Overall N	0.370	0.378	0.407	0.412	0.415	0.390	0.406	0.023	0.72
Overall G	0.952	0.783	0.933	0.887	0.930	0.751	0.894	0.060	0.19
Overall F	1.028	1.077	1.119	1.162	1.096	1.078	1.103	0.031	0.12
Overall	0.820	0.782	0.857	0.858	0.852	0.771	0.837	0.029	0.22

Feces from six pigs with heavier (H) or lighter (L) body weights at the nursery (N), grower (G), and finisher (F) stages were collected and were used as donors for six groups of weaned pigs (NH, NL, GH, GL, FH, and FL). Pigs in the control group didn’t receive FMT from any donors

### Longitudinal fecal microbiota development of the FMT recipients

To examine the effects of early FMT on long-term microbiota dynamics, we sequenced the bacterial 16S rRNA gene V4 region of fecal swab samples collected from 21 to 174 days old recipient pigs from the seven groups together with six donors. After low-quality sequences filtering, we obtained 4,502,748 high-quality reads with an average of 13,727 reads per sample. All sample reads were rarefied to the minimum sequencing depth (5907), which reduced total reads to 1,931,589. These reads were classified into 3411 bacterial ASVs.

All pigs developed a steady increase in microbiota richness and diversity throughout the study, as indicated by chao1, observed_ASV, and Shannon indexes (Fig. S1). The six donor pigs (NLD, NHD, GLD, GHD, FLD, and FHD) presented typical microbial diversities which corresponded to their respective stages (Fig. S2; chao1: 309, 177, 525, 519, 582, and 534; observed_ASV: 279, 172, 439, 436, 523, and 497; Shannon 5.64, 5.29, 6.97, 6.74, 7.30, and 7.12, respectively). The introduction of various age donor fecal microbiota did not significantly affect the overall alpha indexes in the recipients except for the differences between GH (also FL by shannon and observed_ASV and Con by shannon) and FH pigs on d 49 (Shannon: 6.53 ± 0.17 and 5.74 ± 0.17, *P* < 0.01; chao1: 474.0 ± 33.86 and 327.9 ± 33.86, *P* = 0.04; observed_ASV: 396.5 ± 26.07 and 286.7 ± 26.07, *P* = 0.06 for GH and FH, respectively), which was mainly due to the large richness and diversity reduction of the FH group. Despite showing no statistical significance, microbiota from the older donors transiently dwelled in the gut on d 25 resulting in corresponding increases of the alpha observations within each group. This pattern disappeared by the next collection date (Fig. S1).

**Fig. 1:**
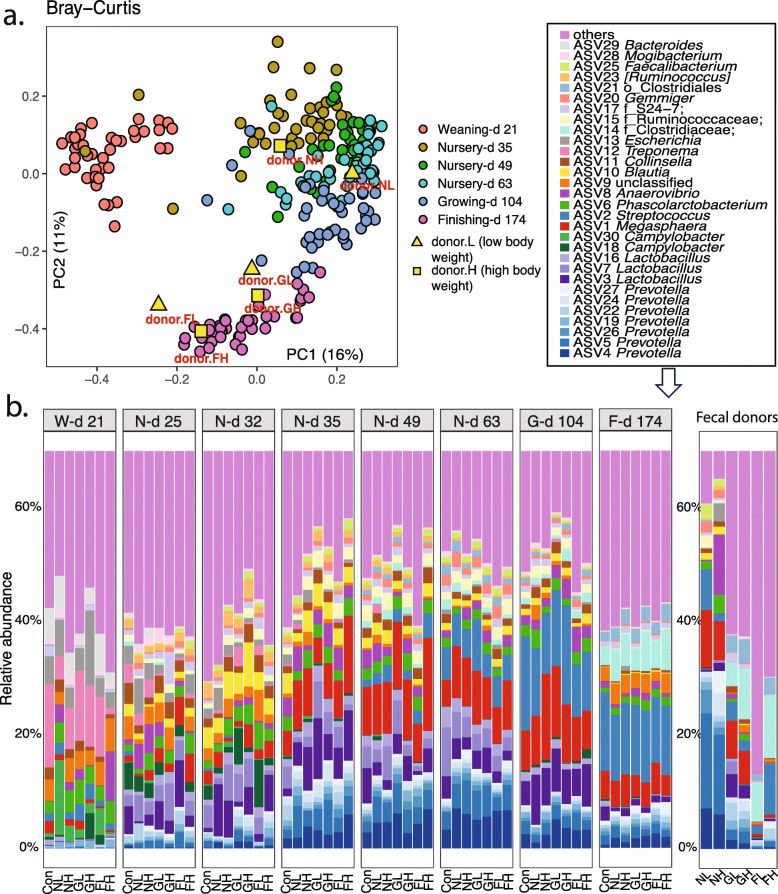
The longitudinal changes of swine gut microbiota structure (Bray-Curtis distance-based PCoA analysis; **a**) and taxonomy composition (top 30 bacterial ASVs, **b**) in recipient pigs gavage fed with PBS with 20% glycerol (Con), fecal microbiota from a nursery pig with low (NL) or high body weight (NH), fecal microbiota from a growing pig with low (GL) or high body weight (GH), and fecal microbiota from a finishing pig with low (FL) or high body weight (FH) at weaning

The principal coordinates analysis (PCoA) based on Bray-Curtis dissimilarity showed an age-driven microbial progression in the recipients from weaning to study completion (Permanova, *P =* 0.001, R = 19.4; Fig. 1a). Distinguishable fecal microbiota structures were observed between sampling days, with the d 21 community exhibiting the most significant discrepancy from the others. The coordinates of the microbiota donors NL, NH, FL, and FH fell within their stage ranges (Fig. 1a and Fig. S2). In contrast, donors GL and GH were close to the recipient microbiota on d 174, resembling an early finishing microbiota type. These beta diversity measures were consistent with the alpha diversity outcome.

The longitudinal taxonomic composition unveiled the reprogramming process of the fecal microbiota from weaning. Significantly different bacterial compositions were observed throughout different growth stages. Specifically, among the top 30 bacterial ASVs, ASV12 *Treponema*, ASV13 *Escherichia*, and ASV29 *Bacteroides* were the most abundant members before weaning when sow milk was the sole diet (Fig. 1b). After weaning and switching to solid diets for five days (d 25), rapid increases in *Lactobacillus* (ASV3, ASV7, and ASV16) and *Prevotella* (ASV4) and decreases in *Treponema* (ASV12) and *Escherichia* (ASV13) were observed to be in agreement with current literature regarding how diet change results in microbiota alteration. From mid-nursery to the growing stage, *Blautia* (ASV10), *Megasphaera* (ASV1), and *Streptococcus* (ASV2) developed into major taxa. At study completion, the relative abundances of *Lactobacillus* and *Blautia* were low, while unclassified (ASV9), *f_Clostridiaceae* (ASV14), and *o_*Clostridiales (ASV21) evolved into predominant bacteria. ASV9 was commonly presented during weaning and early nursery stages but became subsidiary taxa during the late nursery and grower periods. Interestingly, it regained a high presence within the microbiota community at study completion (Fig. 1b). Furthermore, 150 ASVs were identified as stage-associated by performing the top 500 ASVs from the Con groups in LEfSe (Table S3).

### FMT modulates recipient fecal microbiota ecology

**Fig. 2:**
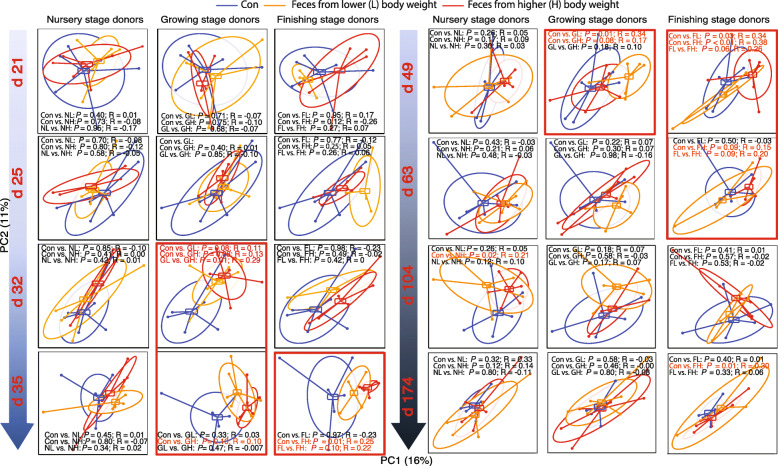
Bray-Curtis distance-based PCoA analysis of longitudinal (d 21, 25, 32, 35, 49, 63, 104 and 174) swine gut microbiota structure in recipient pigs gavage fed with PBS + 20% glycerol (Con) and fecal microbiota from various growth stage donor pigs with either low (L) or high (H) body weight

Bray-Curtis dissimilarity-based ANOSIM was performed throughout the entire course to evaluate the modulation effects on the fecal microbial community (Fig. 2). Pigs gavaged with nursery stage microbiota rarely showed any influence upon the microbiota structures at most time points except d 104 (Con vs. NH: *P =* 0.02; R = 0.21; Fig. 2). However, the microbiota from growing and finishing donors shaped the microbiota of recipients at varying degrees during the nursery stage (Fig. 2). Specifically, the microbiota in both GH and FH received piglets showed detectable changes (Con vs. GH: *P =* 0.10; R = 0.10; Con vs. FH: *P =* 0.01; R = 0.25) on d 35. On d 49, pigs administrated with growing and finishing microbiota (regardless of BW criteria) at weaning displayed altered fecal microbiota structures (*P* < 0.10 and R = 0.17–0.38 for all comparisons between Con and GL, GH, FL, and FH). FMT of finishing stage microbiota from a heavy donor resulted in long-lasting effects on the fecal microbiota community in the recipients, as indicated by the ANOSIM, Con vs. FH: *P =* 0.09 (R = 0.15) and 0.01 (R = 0.30) on d 63 and d 174, respectively (Fig. 2). Noteworthy is that microbiota from the higher BW donors modulated, to a greater degree, the recipient fecal microbiota than those from the lower BW counterparts regardless of stage.

### Contributions of FMT to recipient fecal microbiota

**Fig. 3:**
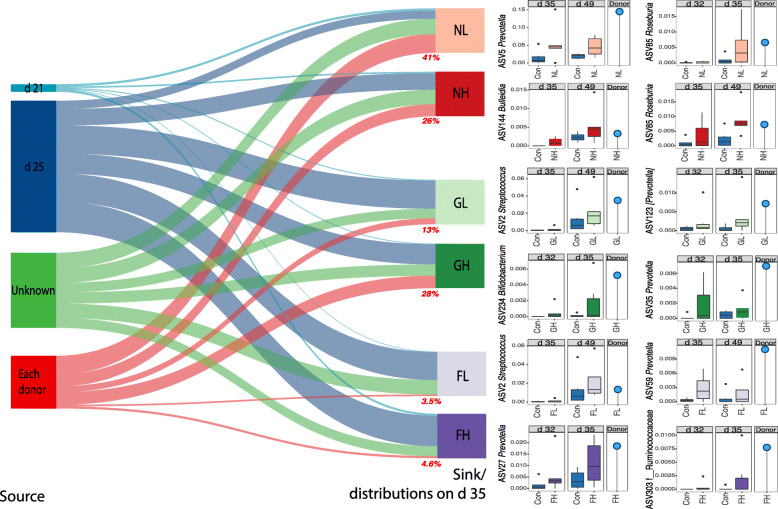
Source contributions to the recipient pig gut microbiota on d 35. SourceTracker was used to calculate the contributions of each source (microbial community at d 21, d 25, and the donors’ microbiota) to the sink (recipient microbiota at d 35) in each transplantation model. The abundances of potential colonizers are shown in the box plots from the donor, control group, and FMT group at d 32/35, or d 35/49

After observing the modulating effects of FMTs, our next step was to study if these changes were due to colonization of the transplanted donor microbiotas. SourceTracker software was used to calculate the contributions of each source (e.g., donors microbiota, fecal microbiota from previous time points) to the sink (the recipient fecal microbiota on specific dates). We chose the microbial communities from d 21 (weaning), d 25 and the microbial donor as the three sources. The d 35 recipient microbiota from each group was used as a sink or the outcome of FMT. Interestingly, the NL and NH donors contributed sizeable percentages (average 41% for NL and 26% for NH) to the community on d 35 in recipient pigs (Fig. 3). Growing stage microbiota contributed smaller proportions to the sinks than from the nursery stage (GL at 13% and GH at 28%). The finishing donors had the least contributions to their recipients, with only 3.5% and 4.6% for FL and FH, respectively. These results suggested that the modulated communities in the recipients were consequences derived from the colonization of only a small group of microbiota members from older donors. Although influencing the microbiota development, most adult donor microbiotas found it very difficult to colonize within the young pigs (Fig. 3).

We next investigated the donors bacterial taxa that possibly colonized or multiplied in the recipient pigs. We found that ASV303 *f_Ruminococcaceae* (FH), ASV234 *Bifidobacterium* (GH), ASV2 *Streptococcus* (GL and FL), and several ASVs of *Prevotella* (ASV5, ASV123, ASV35, ASV59, and ASV27) were potential colonizers (Fig. 3). We further estimated the biological ages of the fecal microbiota in the recipient pigs using regression-based random forest, a machine learning-based maturity indicator [31]. The non-FMT group samples from all time points were used to establish the age model, which is based on 50 of most age-associated ASVs identified by random forest (Fig. S3). As expected, all FMT pigs using older donor microbiota achieved improved EMA numerically on d 35 (35.9, 41.8, 47.9, 41.4, 43.7, 42.5, and 42.9 for Con, NL, NH, GL, GH, FL, and FH, respectively; *P* = 0.19) and 49 (50.3, 53.5, 53.1, 51.5, 53.7, 59.6, and 55.8, respectively; *P* = 0.08). However, for most groups, this effect faded by the end of the nursery stage (Fig. S4a and b). Specifically, ASV206 *Ruminococcaceae*, ASV211 *Butyrivibrio*, ASV416 *Bacteroides*, ASV53 *[Paraprevotellaceae]*, and ASV182 *Oscillospira* potentially drove the fecal microbiota maturation on d 35, and ASV2 *Streptococcus*, ASV247 *Lachnospiraceae*, ASV291 *Veillonellaceae*, ASV47 *Mitsuokella*, and ASV32 *Dialister* were possibly linked to the expedited maturity on d 49 (Fig. S4c). Not surprisingly, some of these EMA-associated features are also age-associated such as ASV2 (d 104) and ASV47 (d 63).

### FMT modulates BW-associated bacteria

**Fig. 4:**
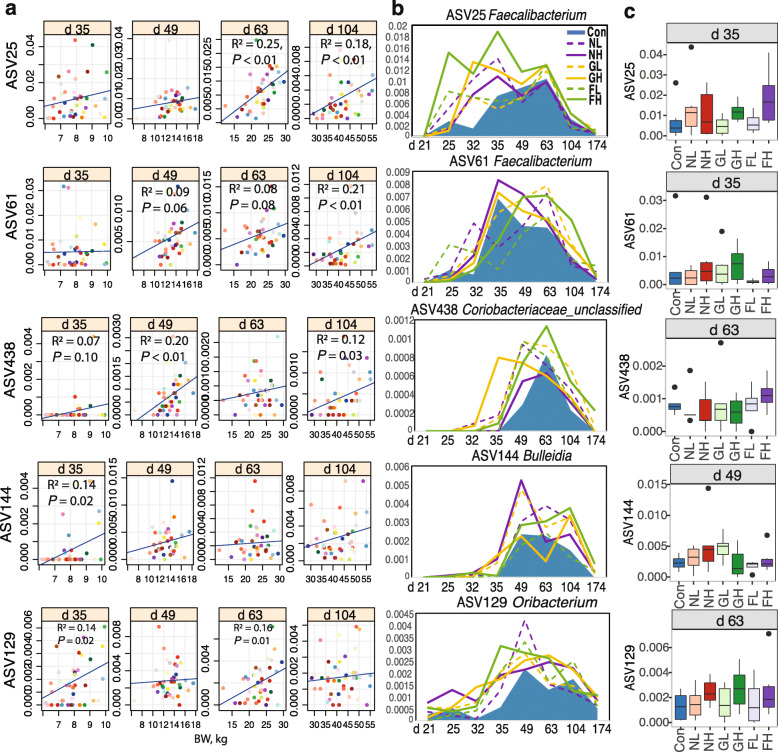
**a**. Scatter plots with regression line showing correlations of bacterial ASVs with body weight (BW, x-axis) on each day. **b**. shows the dynamics of these bacterial ASVs from different groups (e.g. control plus six FMTs). **c**. Box color represents the relative abundances of these ASVs on specific days (Con/blue: control group; NL/light red: FMT from a light BW donor at the nursery stage; NH/dark red: FMT from a heavy BW donor at the nursery stage; GL/light green: FMT from a light BW donor at the growing stage; GH/dark green: FMT from a heavy BW donor at the growing stage; FL/light red: FMT from a light BW donor at the finishing stage; FH/dark red: FMT from a heavy BW donor at the finishing stage)

Five potential BW boosters (ASV25 *Faecalibacterium*, ASV61 *Faecalibacterium*, ASV438 *Coriobacteriaceae*_unclassified, ASV144 *Bulleidia*, and ASV129 *Oribacterium*) positively related to BW were identified on d 35, 49, 63, and 104 (Fig. 4a). To note, these bacterial members were enriched by FMT, especially by the heavier donors such as NH, GH, and FH (Fig. 4b and c). Furthermore, the microbiota transplantation lengthened the dominant duration of these bacteria by either advancing the exponential growth phase (ASV25, ASV438, and ASV129) or extending the decline phase (ASV61 and ASV144). Age-associated BW boosters were also isolated. At the end of the nursery stage (d 63), ASV353 *f_Lachnospiraceae*, ASV5 *Prevotella*, and ASV8 *Anaerovibrio* that were positively correlated to BW, were generally more abundant in recipients administered with heavier donor microbiota than other groups (Fig. S5a). At the end of growing phase 1 (d 104), abundances of ASV2 *Streptococcus*, ASV14 *f_Clostridiaceae*, and ASV136 *Blautia* (Fig. S5a) were BW-associated. Different groups of beneficial bacterial members such as *Veillonellaceae*, *Ruminococcaceae*, *Faecalibacterium*, and *Succinivibrio* were observed to be modulated in the early (d 35) and middle (d 49) nursery stages (Fig. S5b and c).

**Fig. 5:**
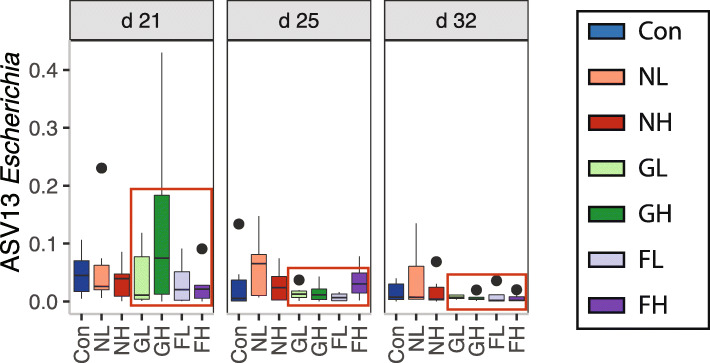
The relative abundance of *E.coli* on d 21, 25, and 32 from each treatment group

Gavage of growing and finishing fecal microbiota resulted in the reduction of *Escherichia* (Fig. 5). However, this reduction was not observed in either NL or NH piglets.

### Microbiotas’ metabolic capability

**Fig. 6:**
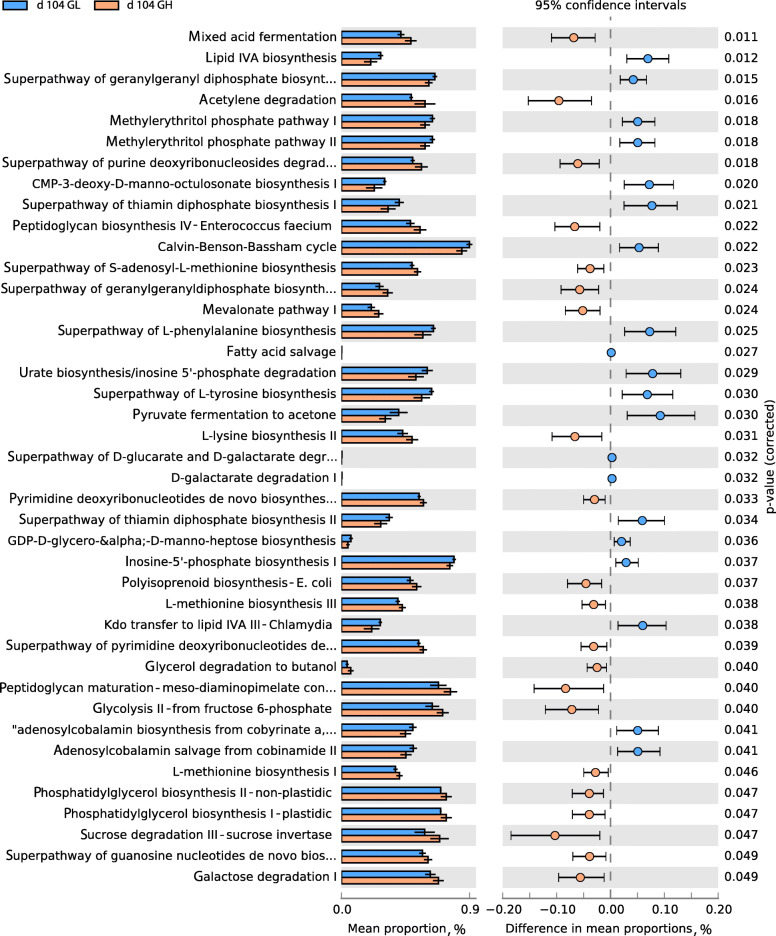
Abundances of the predicted metabolic pathways in the recipient gut microbiotas from the light (GL) and heavy body weight donors (GH) on d104

We compared the metabolic activities in fecal microbiotas between GH pigs and those in the Con and GL groups to understand the underlying mechanisms that potentially led to improved growth performances. We initially evaluated their metabolic functions on d 104, when the GH pigs gained the highest BW (42.1, 41.7, and 47.1 kg for Con, GL, and GH, respectively). The metabolic pathways of pyridoxal 5′-phosphate biosynthesis and tRNA processing were greatly reduced in GH groups compared to their Con counterparts (Fig. S6). When compared to GL pigs, microbiota from a heavier donor increased the synthesis of essential amino acids lysine and methionine, mixed acid fermentation along with enhanced glycolysis, and sucrose/galactose degradation. The pathway of inosine-5′-phosphate biosynthesis, a subclass of nucleoside and nucleotide biosynthesis, possibly possessed by *Escherichia K-12* and *Salmonella* was also reduced in GH pigs (Fig. 6). We then investigated an earlier time point d 49 when distinguishable communities were observed in GL and GH pigs and found the abundances of the biosynthesis of amino acids (methionine and three branched chain amino acids) were consistently higher in GH pigs than the GL counterparts. However, certain carbohydrate metabolism related pathways such as glycolysis and galactose degradation, and mixed acid fermentation were reduced in GH pigs on d 49, which were different from those on d 104 (Fig. S7). Abundances of these glycolytic pathways (I, II, and III) in GH pigs were also lower than the Con group on d 49. However, amino acids (tyrosine, phenylalanine, and branched-chain amino acids) synthesis pathways were enriched in GH groups when compared to Con (Fig. S8).

## Discussion

Weaning brings about the most profound reprogramming of the gut microbiota in pigs. During this crucial period, piglets are highly susceptible to extraneous pathogens such as enterotoxigenic *E. coli*, *Salmonella choleraesuis*, and *Campylobacter*, which can cause severe diarrhea resulting in increased morbidity and mortality rates. Strategies dealing with this critical problem are usually accomplished through microbiota interventions, such as administrations of bactericidal agents (antibiotics, minerals, essential oils or organic acids), probiotics (lactic acid bacteria, *Bacillus*, and yeast), and prebiotics (oligofructose, fructooligosaccharide, mannose oligosaccharide, and inulin) [3, 32–36]. In this study, fecal microbiota transplantations, previously discovered as a cure to treat *Clostridioides difficile* infection in humans, were investigated for their effects on growth, fecal microbiota succession, ecology, and metabolic potentials in pigs.

Due to the small sample size, the recipient’s growth performance was not influenced significantly. However, a clear pattern was observed that donor BW and stage have varying effects on the final BW of recipients across different donor stages. During the nursery stage, all microbiota transplanted pigs (NL, NH, GL, GH, FL, and FH) achieved improved BW numerically when compared to the Con. This finding was partially supported by our previous study; gavage-fed weaned piglets with growing stage microbiota elevated both short- and long-term ADG and BW [21, 37]. In another study, FMT from an adult pig into newborn piglets improved their ADG by d 28 and significantly reduced diarrhea incidences and pathogen shedding [38]. However, different growth effects were observed between the pigs that received heavier and lighter donors during the growing stage. Specifically, recipients who received microbiota from heavy donors had heavier body weight numerically than pigs gavaged with bacteria from their lighter counterparts. Noteworthy, the growth of pigs fed low body weight donor microbiotas was slower than the pigs in the control group. This pattern continued until the end of the finishing stage for all groups except those administered with growing stage microbiotas, of which similar growth-promoting effects were observed. Not surprised, deleterious effects were reported in offspring intestine histology and growth performance, when pregnant sows were inoculated with highly feed-efficient pigs’ gut microbiotas. This together with our observation, emphasized the importance of screening a donor’s background to prevent the introduction of pathogenic bacteria such as *Spirochaetes* and *Chlamydiae* to receipients. Collectively, studies comparing different donor effects are still lacking, and our study provided preliminary insights on how donor stage and phenotypes contributed to the growth effects of the recipients, which emphasized the donor selection in FMT.

The polarized growth effects from light and heavy donors suggested that the benefits of phenotypic traits can be transferred to the recipients through FMT. We found that the modulation effects of FMT on the recipients’ microbiota communities are related to the donors’ stage and growth performance: (1) microbiota from finisher stage donors had greater influence than those from grower stage on weaning recipients, (2) influence from nursery stage donor microbiota upon recipients was rarely observed, and (3) microbiota from higher BW donors (GH and FH) influenced, to a greater degree, the recipient fecal community compared to their lower BW counterparts (GL and FL). This data aligns with other studies that feeding adult pigs’ fecal microbiota to postnatal piglets significantly improved their fecal microbial structure [39–41]. Not surprisingly, the oldest donor exerted the most profound effect on the recipient microbiome due to a greater difference between young and adult pig microbiotas. To note, the modulation effects by FMT were not discernible until ten days after gavage, which is also comparable with a previous study with a two-week adaptation [21].

Diet is a major driver for longitudinal fecal microbiota development [21, 42]. Substantial changes occurred to their fecal community when pigs transitioned from easily digestible high protein sow milk to a high fiber diet containing high indigestible polysaccharides. An adaptation of the microbial community is necessary when faced with such a drastic dietary change to maintain the host energy and essential nutrient requirements [43]. Interestingly, we estimated the biological ages of the microbiota and found that FMT from older donors transiently expedited the microbial succession in the recipients, which were potentially linked to the physiological characteristics (i.e. digestion and immune capability) of the host [31, 44]. Many taxa known to digest fiber and complex polysaccharides such as *Prevotella*, *Lactobacillus*, *Streptococcus*, *Ruminococcus*, *Butyrivibrio*, *Bacteroides,* and *Blautia*, were identified as the most stage-associated microbiota members. Specifically, *Prevotella*, *Bacteroides*, and *Ruminococcaceae_*unclassified potentially encoding a variety of carbohydrate-active enzymes, including polysaccharide lyases, were remarkably enriched during early postweaning [45, 46] to benefit host digestion. In a previously published study, microbiota maturity was used to measure the postnatal malnourished states in Bangladeshi children. Resembling our findings, *Faecalibacterium*, *Ruminococcus*, *Streptococcus,* and *Ruminococcaceae_*unclassified were the top bacterial taxonomic biomarkers for healthy and maturated microbiota in these children during the first two years of life [47]. Hence, these enriched features, together with community maturity, are directly linked to host health during early life.

The advanced succession of the microbiota might be attributed to the potential colonization of bacteria from donors after weaning. We then evaluated the contributions of donor microbial populations to the recipient microbiotas on d 35 using SourceTracker. Unsurprisingly, nursery microbiotas are the most adaptable to the recipients’ gut environments. Hence, colonizing efficiency of the microbiota is related to donor growth stage, which coincided with the previous discovery that swine gut microbiotas are stage-associated [21]. The low colonization rate of older donor microbiota in the recipients could be due to the considerable differences in diet compositions and gut physiological environments between the donors and recipients [48]. When sorting out the potential colonizers in the post-weaned recipients, we observed a substantial amount of *Prevotella*, *Streptococcus*, *Ruminococcaceae_*unclassified*,* and *Roseburia* that greatly amplified two weeks after gavage. Noteworthy, these bacteria possess robust abilities to digest complex carbohydrates, which explains the rapid colonization after diet transition [49, 50]. Besides, among these colonizers, ASV2 *Streptococcus*, ASV144 *Bulleidia*, ASV234 *Bifidobacterium*, and ASV59 *Prevotella* also belonged to the top 50 EMA-associated feature list, which contributed to the microbiota succession.

Studies using FMT to modulate piglet health and production remain rare and sporadic. For the first time, our study demonstrated the effects of FMT from various growth stages and phenotype donors on piglet fecal microbiota development and growth performance. We hypothesized that the increased BW of the recipients was linked to the enrichment of certain beneficial or commensal bacteria. Interestingly, we found the abundance of some ASVs within *Faecalibacterium*, *Coriobacteriaceae_*unclassified, *Bulleidia,* and *Oribacterium* were positively correlated to the BW during nursery and early growing stage. Generally, these bacteria were more abundant in pigs administered with heavier donor microbiotas than the lighter counterparts. Hence, these taxa might contribute to the different growth effects of FMT between heavier and lighter BW donors. Not surprisingly, *Faecalibacterium* is a symbiotic microorganism that has been considered as a probiotic bacterium to improve both human and swine gut health by suppressing proinflammation, improving barrier functions, and producing butyrate [51–54]. The low abundance of this bacteria is associated with metabolic deficiency-induced gut diseases such as inflammatory bowel disease and Crohn’s disease [55, 56]. *Coriobacteriaceae* was reported as a potential feed efficiency booster, and *Bulleidia* was inversely associated with diarrhea incidences in piglets and a butyrate producer [57–59]. The biological functions of *Oribacterium* have rarely been reported, but this bacterium was highly presented in sows with high productivity [60]. Of note, not only the abundance but also the duration of these beneficial bacteria were promoted by FMT in the recipients. Other bacterial ASVs that were positively associated with increased nursery stage BW of pigs receiving grower and finisher microbioita included ASV63 *Catenibacterium*, ASV396 *f_Ruminococcaceae*, ASV32 *Dialister*, and ASV76 *Succinivibrio. Catenibacterium* is a gram-positive, obligatory anaerobe that can produce various short-chain fatty acids (acetic, lactic, butyric, and iso-butyric acids) that are known to support epithelium maintenance [61]. *Succinivibrio* has been reported to be one of the major core swine gut microbial members highly involved with carbohydrate degradation [62–64]. On d 63 and 104, BW-associated taxa *Blautia*, *Streptococcus*, and *Prevotella* were generally higher in pigs fed microbiota from heavier donors than lighter ones. These bacterial members have been widely studied and described as dominant commensal or beneficial bacteria in swine [21, 53, 65, 66].

Besides the upregulation of beneficial bacteria, the *Escherichia* abundance was reduced in the GL, GH, FL, and FH recipients during early nursery stage. However, contrary outcomes were observed in another study where newborn piglets showed a reduction in growth performance, intestinal histology, and increased pathogens such as *Campylobacter* after transplanting four doses of finishing stage fecal microbiota from high feed efficiency donors [67]. Other adverse effects were stimulated proinflammatory responses, reduced serum protein production and decreased cholesterol concentrations which suggested that gut dysbiosis was a result of the FMT. This outcome could be due to the failure of screening for a donor candidate absent of pathogens. Hence, proper donor selection is critically essential for FMT [25].

We further investigated the metabolic functions of the microbiotas in Con, GL, and GH pigs on d 104 and 49 to understand how they contributed to BW gain. The improved BW of GH pigs was possibly linked to a higher abundance of various amino acid producing bacteria at both time points compared to the GL group. This is especially true regarding bacteria that produce the branched-chain amino acids and essential amino acids such as methionine and lysine. Branched-chain amino acids serve as regulators of energy homeostasis in muscle, nutrition metabolism, gut health, and immune functions in pigs [68]. Also, they act as signaling molecules that stimulate pathways related to cell growth and protein synthesis, such as phosphoinositide 3-kinase/protein kinase B/mammalian target of rapamycin (PI3K/AKT/mTOR) [69]. Lysine and methionine are the top two essential amino acids for swine. Their importance to muscle protein deposition and immune-related protein synthesis has been emphasized in many studies. A shortage of lysine and methionine in immune-activated pigs will cause increased muscle protein catabolism [70]. The small intestine has been known as the main amino acid absorption site but the amino acid uptake from hindgut should not be overlooked. Metges found that fecal microbial lysine represents 5% to 7% of host plasma lysine pool [71]. This suggested that these additional microbial amino acids in GH may have benefitted the growth performance observed on d 104.

## Conclusion

We examined the effect of donor growth stages and phenotypes on weaning piglet microbiota development and growth performance by FMT. Growth stage donors possessed the greatest growth booster effect numerically compared to nursery and finishing donors. Also, the growth phenotypes could be transmitted to the recipients through FMT, especially during nursery and growing stages. This benefit could be attributed to the enrichments of ASV25 *Faecalibacterium*, ASV61 *Faecalibacterium*, ASV438 *Coriobacteriaceae_*unclassified, ASV144 *Bulleidia*, and ASV129 *Oribacterium* and reduction of ASV13 *Escherichia* during the nursery stage. The growing and finishing donor materials significantly influenced the community for a short-term during the nursery stage. Furthermore, older donor microbiota noticeably expedited maturation on d 35 and 49, as indicated by EMA and the enrichments of EMA-associated bacterial ASVs*.* The fecal metabolic activities of microbiotas resembling the lower GI tract potentially benefit the host by modulating essential amino acid synthesis and energy processing. Our study elucidated the beneficial use of FMT regarding growth, microbiota community shifts, ecological succession, and metabolic potentials. Also, our study can serve as a donor selection guide for maximizing the positive outcomes of FMT use in swine.

## Supplementary Information

**Additional file 1: Table S1.** Nursery diet composition (as fed). **Table S2.** Growing and finishing diet composition (as fed). **Table S3.** List of age-associated bacterial features identified by LEfSe. **Fig. S1.** Longitudinal dynamics of alpha diversities (chao1, observed_features, and Shannon) of the gut microbiota in recipient pigs gavage fed with PBS with 20% glycerol (Con), fecal microbiota from a nursery pig with low (NL) or high body weight (NH), fecal microbiota from a growing pig with low (GL) or high body weight (GH), and fecal microbiota from a finishing pig with low (FL) or high body weight (FH) at weaning (a). Alpha diversities (chao1, observed_features, and shannon) of the six donors (b). **Fig. S2.** Bray-Curtis dissimilarity-based PCOA plots of gut microbial communities from donors and recipient pigs on d25, 35, 49, 63, and 174. Points represent the gut microbiota in recipient pigs gavage fed with PBS with 20% glycerol (Con/yellow), fecal microbiota from a nursery pig with low (NL/light red) or high body weight (NH/dark red), fecal microbiota from a growing pig with low (GL/light green) or high body weight (GH/dark green), and fecal microbiota from a finishing pig with low (FL/light purple) or high body weight (FH/dark purple) at weaning (a). Donors are yellow color filled triangles (light BW) and squares (heavy BW). **Fig. S3.** The top 50 estimated age (EMA)-associated features identified by the regression-based random forest. **Fig. S4.** Boxplots present the estimated microbiota ages (EMA) of the gut microbiota in recipient pigs on d 32, 35, 49, 63, and 104 (a). Distributions of EMA values from each group on d35 and 49 (b). The relative abundances of potential modulated bacterial members associated with EMA on d35 and 49 by fecal microbial transplantations (c). **Fig. S5.** Boxplots showing the relative abundances of BW-associated bacterial features at each treatment (a. d63 and 104; b. d35; c. d49). The scatter plots with regression line showing correlations of these features with body weight (BW) on each day. Box color represents different FMT groups (Con/blue: vehicle group; NL/light red: nursery stage microbiota from a light BW donor; NH/dark red: nursery stage microbiota from a heavy BW donor; GL/light green: growing stage microbiota from a light BW donor; GH/dark green: growing stage microbiota from a heavy BW donor; FL/light red: finishing stage microbiota from a light BW donor; FH/dark red: finishing stage microbiota from a heavy BW donor). **Fig. S6.** Abundances of the predicted metabolic pathways in the recipient fecal microbiotas that were gavage fed either a placebo or growing stage microbiotas from a heavy body weight donor (GH) on d104. **Fig. S7.** Abundances of the influenced metabolic pathways predicted in the recipient microbiotas that were gavage fed growing stage microbiotas from both light (GL) and heavy body weight donors (GH) on d49. **Fig. S8.** Abundances of the influenced metabolic pathways predicted in the recipient microbiotas that were gavage fed either placebo or growing stage microbiotas from a heavy body weight donor (GH) on d49.

## Data Availability

The datasets can be found in the Sequence Read Archive database, BioProject accession number is PRJNA722740.

## Abbreviations

ASV: Amplicon sequence variant
FMT: Fecal microbial transplantation
IACUC: Institutional Animal Care and Use Committee
BW: Body weight
NH: Heavy BW donor from nursery stage
NL: Light BW donor from nursery stage
GH: Heavy BW donor from grower stage
GL: Light BW donor from grower stage
FH: Heavy BW donor from finisher stage
FL: Light BW donor from finisher stage
ADG: Average daily gain
PCR: Polymerase chain reaction
PERMANOVA: Permutational multivariate analysis of variance
ANOSIM: Analysis of similarity
PICRUSt2: Phylogenetic Investigation of Communities by Reconstruction of Unobserved States 2
KEGG: Kyoto Encyclopedia of Genes and Genomes
STAMP: Statistical Analysis of Metagenomic Profiles
PCoA: Principal coordinates analysis
EMA: Estimated ages
