# Supplementary material for: Donor age and body weight determine the effects of fecal microbiota transplantation on growth performance, and fecal microbiota development in recipient pigs
Source: J Anim Sci Biotechnol. 2022 Apr 11;13:49. doi: 10.1186/s40104-022-00696-1 (PMC8996565; doi:10.1186/s40104-022-00696-1)
Supplement: Supplementary file 1 — Additional file 1: Table S1. Nursery diet composition (as fed). Table S2. Growing and finishing diet composition (as fed). Table S3. List of age-associated bacterial features identified by LEfSe. Fig. S1. Longitudinal dynamics of alpha diversities (chao1, observed_features, and Shannon) of the gut microbiota in recipient pigs gavage fed with PBS with 20% glycerol (Con), fecal microbiota from a nursery pig with low (NL) or high body weight (NH), fecal microbiota from a growing pig with low (GL) or high body weight (GH), and fecal microbiota from a finishing pig with low (FL) or high body weight (FH) at weaning (a). Alpha diversities (chao1, observed_features, and shannon) of the six donors (b). Fig. S2. Bray-Curtis dissimilarity-based PCOA plots of gut microbial communities from donors and recipient pigs on d25, 35, 49, 63, and 174. Points represent the gut microbiota in recipient pigs gavage fed with PBS with 20% glycerol (Con/yellow), fecal microbiota from a nursery pig with low (NL/light red) or high body weight (NH/dark red), fecal microbiota from a growing pig with low (GL/light green) or high body weight (GH/dark green), and fecal microbiota from a finishing pig with low (FL/light purple) or high body weight (FH/dark purple) at weaning (a). Donors are yellow color filled triangles (light BW) and squares (heavy BW). Fig. S3. The top 50 estimated age (EMA)-associated features identified by the regression-based random forest. Fig. S4. Boxplots present the estimated microbiota ages (EMA) of the gut microbiota in recipient pigs on d 32, 35, 49, 63, and 104 (a). Distributions of EMA values from each group on d35 and 49 (b). The relative abundances of potential modulated bacterial members associated with EMA on d35 and 49 by fecal microbial transplantations (c). Fig. S5. Boxplots showing the relative abundances of BW-associated bacterial features at each treatment (a. d63 and 104; b. d35; c. d49). The scatter plots with regression line showing correlations of these fe [file 40104_2022_696_MOESM1_ESM.docx]

Table S1. Nursery diet composition (as fed).

|  | Phase 1 | Phase 2 | Phase 3 |
| --- | --- | --- | --- |
| Ingredients, % |  |  |  |
| Corn | 38.8 | 44.5 | 47.5 |
| Soybean meal, 48% | 25 | 31.6 | 31.6 |
| Corn DDGS, >6 and <9% Oil | 0 | 15 | 15 |
| Poultry Fat | 3 | 3 | 3 |
| Monocalcium P | 0.35 | 0.3 | 0.25 |
| Limestone | 0.7 | 1.05 | 1.15 |
| L-Lysine | 0.22 | 0.36 | 0.36 |
| DL-Methionine | 0.175 | 0.135 | 0.114 |
| L-Threonine | 0.0525 | 0.08 | 0.066 |
| L-Tryptophan | 0 | 0.0025 | 0 |
| Trace Mineral Premix^1^ | 0.15 | 0.15 | 0.15 |
| Vitamin Premix^2^ | 0.25 | 0.25 | 0.25 |
| Plasma | 2.5 | 0.5 | 0 |
| Fish Meal | 5 | 2.5 | 0 |
| Milk, Whey Powder | 20 | 0 | 0 |
| Milk, Lactose | 3.5 | 0 | 0 |
| Others^3^ | 0.295 | 0.545 | 0.545 |
| Total | 100 | 100 | 100 |
| Calculate: |  |  |  |
| ME^4^, kcal/kg | 3482 | 3438 | 3434 |
| CP^4^, % | 22.91 | 25.37 | 23.64 |
| SID^4^ Lysine, % | 1.46 | 1.43 | 1.29 |
| Aval. P (%) with phytase | 0.55 | 0.41 | 0.32 |
| Ca, % | 0.85 | 0.75 | 0.65 |
| SID M+C:Lys | 58.28 | 58.02 | 58.10 |
| SID Thr:Lys | 60.09 | 60.20 | 60.05 |
| SID Trp:Lys | 18.42 | 17.95 | 18.18 |
| SID Ile:Lys | 59.77 | 62.96 | 64.66 |
| SID Val:Lys | 66.81 | 70.17 | 71.32 |
| ^1^The mineral premix provided the following per kg of complete diet: 84 mg of Ca, 165 mg of Fe, 165 mg of Zn, 39.6 mg of Mn, 16.5 mg of Cu, 0.3 mg of I, and 0.3 mg of Se.  ^2^The vitamin premix provided the following per kg of complete diet: 485 mg of Ca, 4133.6 IU of vitamin A, 1653.4 IU of vitamin D_3_, 44.09 IU of vitamin E, 0.0331 mg vitamin B_12_, 3.31 mg of menadione, 8.27 mg of riboflavin, 27.56 mg of D-pantothenic acid, and 49.6 mg of niacin.  ^3^Others contained sodium chloride (0.25%, 0.5%, 0.5% in phase 1, 2, and 3, respectively), phytase (0.015%), antioxidant (0.03%), and titanium dioxide (0.15% in phase 2 and 3).  ^4^ME – metabolizable energy; CP – crude protein; SID – standard ileal digestible. | | | |

Table S2. Growing and finishing diet composition (as fed).

|  | Growing 1 | Growing 2 | Finishing 1 | Finishing 2 |
| --- | --- | --- | --- | --- |
| Ingredients, % |  |  |  |  |
| Corn | 61.72 | 68.92 | 73.17 | 75.82 |
| Soybean meal (48%) | 20.75 | 13.6 | 9.6 | 7 |
| Corn DDGS | 15 | 15 | 15 | 15 |
| Monocalcium phosphate | 0.245 | 0.225 | 0.17 | 0.15 |
| Limestone | 1.16 | 1.175 | 1.125 | 1.05 |
| Sodium chloride | 0.35 | 0.35 | 0.35 | 0.35 |
| Trace Mineral Premix^1^ | 0.15 | 0.15 | 0.1 | 0.1 |
| Vitamin Premix^2^ | 0.15 | 0.15 | 0.125 | 0.125 |
| L-Lysine | 0.35 | 0.325 | 0.284 | 0.3 |
| L-Threonine | 0.057 | 0.043 | 0.024 | 0.047 |
| DL-Methionine | 0.012 | - | - | - |
| L-Tryptophan | 0.0085 | 0.013 | 0.0115 | 0.017 |
| Ronozyme P CT | 0.015 | 0.015 | 0.015 | 0.015 |
| Ethoxiquin^3^ | 0.03 | 0.03 | 0.03 | 0.03 |
| Calculate: |  |  |  |  |
| ME^4^, kcal/kg | 3307 | 3314 | 3324 | 3330 |
| CP^4^, % | 19.56 | 16.70 | 15.08 | 14.09 |
| SID^4^ Lysine, % | 1.02 | 0.82 | 0.69 | 0.64 |
| Available P (%) with phytase | 0.30 | 0.28 | 0.26 | 0.25 |
| Ca, % | 0.61 | 0.59 | 0.54 | 0.50 |
| SID M+C:Lys | 55.05 | 58.98 | 65.04 | 66.65 |
| SID Thr:Lys | 61.06 | 62.05 | 63.09 | 66.11 |
| SID Trp:Lys | 18.05 | 18.05 | 18.05 | 18.09 |
| SID Ile:Lys | 64.55 | 65.29 | 67.92 | 66.56 |
| SID Val:Lys | 73.73 | 77.04 | 82.17 | 82.13 |
| SID Leu:Lys | 155.65 | 172.09 | 191.01 | 196.72 |
| SID His:Lys | 44.02 | 46.17 | 49.38 | 49.46 |
| ^1^The mineral premix provided the following per kg of complete diet: 84 mg of Ca, 165 mg of Fe, 165 mg of Zn, 39.6 mg of Mn, 16.5 mg of Cu, 0.3 mg of I, and 0.3 mg of Se.  ^2^The vitamin premix provided the following per kg of complete diet: 291 and 194 mg of Ca, 2480.2 and 1653.4 IU of vitamin A, 992.1 and 661.4 IU of vitamin D_3_, 26.46 and 17.64 IU of vitamin E, 0.0198 and 0.0132 mg vitamin B_12_, 1.98 and 1.32 mg of menadione, 4.96 and 3.31 mg of riboflavin, 16.53 and 11.02 mg of D-pantothenic acid, and 29.76 and 19.84 mg of niacin for grower and finisher phases, respectively.  ^6^Quinguard, Novus International, Inc., St. Louis, MO.  ^7^ME – metabolizable energy; CP – crude protein; SID – standard ileal digestible. | | | | |

Table S3. List of age-associated bacterial features identified by LEfSe.

| **d 21** | **d 35** | **d 63** | **d 174** |
| --- | --- | --- | --- |
| ASV12 Treponema | ASV10 Blautia | ASV7 Lactobacillus | ASV14 f_Clostridiaceae; |
| ASV13 Escherichia coli | ASV8 Anaerovibrio | ASV4 Prevotella copri | ASV31 YRC22 |
| ASV9 unclassified | ASV22 Prevotella | ASV5 Prevotella copri | ASV39 Phascolarctobacterium |
| ASV40 Oscillospira | ASV24 Prevotella | ASV16 Lactobacillus reuteri | ASV56 Clostridium |
| ASV11 Collinsella aerofaciens | ASV23 [Ruminococcus] | ASV20 Gemmiger formicilis | ASV21 o_Clostridiales |
| ASV35 Prevotella | ASV36 [Prevotella] | ASV34 Lactobacillus | ASV66 Turicibacter |
| ASV28 Mogibacterium | ASV43 Prevotella | ASV25 Faecalibacterium prausnitzii | ASV59 Prevotella |
| ASV224 f_[Mogibacteriaceae] | ASV269 unclassified | ASV45 Blautia | ASV111 Prevotella |
| ASV130 f_Ruminococcaceae | ASV140 [Prevotella] | ASV47 Mitsuokella | ASV60 f_Peptostreptococcaceae |
| ASV94 Oscillospira | ASV54 Succinivibrio | ASV160 f_Ruminococcaceae | ASV75 f_Lachnospiraceae |
| ASV58 [Eubacterium] | ASV61 Faecalibacterium | ASV150 o_Bacteroidales | ASV77 Prevotella |
| ASV257 Oscillospira | ASV64 f_Ruminococcaceae | ASV122 f_Coriobacteriaceae | ASV125 Prevotella |
|  | ASV38 Roseburia | ASV63 Catenibacterium | ASV190 unclassified |
| **d 49** | ASV50 CF231 | ASV229 Peptococcus | ASV139 Treponema |
| ASV3 Lactobacillus | ASV112 f_Ruminococcaceae | ASV100 Prevotella | ASV147 Mitsuokella |
| ASV15 f_Ruminococcaceae; | ASV82 Anaerovibrio | ASV124 f_Coriobacteriaceae | ASV87 f_Ruminococcaceae |
| ASV42 Prevotella | ASV67 Dorea | ASV490 f_Coriobacteriaceae | ASV346 f_[Mogibacteriaceae] |
| ASV26 Prevotella copri | ASV76 Succinivibrio | ASV211 Butyrivibrio | ASV275 f_Ruminococcaceae |
| ASV51 Prevotella | ASV37 o_Bacteroidales | ASV212 Slackia | ASV369 Peptoniphilus |
| ASV53 f_[Paraprevotellaceae] | ASV180 f_Ruminococcaceae | ASV345 Lactobacillus | ASV134 f_Lachnospiraceae |
| ASV70 Blautia | ASV200 [Ruminococcus] | ASV356 f_Coriobacteriaceae | ASV252 f_Ruminococcaceae |
| ASV84 Peptococcus | ASV92 Coprococcus | ASV171 f_Ruminococcaceae | ASV103 Prevotella |
| ASV49 f_Lachnospiraceae | ASV157 Streptococcus | ASV137 f_Coriobacteriaceae | ASV202 Lactobacillus |
| ASV68 [Prevotella] | ASV193 Mitsuokella | ASV344 Coprococcus | ASV230 f_[Paraprevotellaceae] |
| ASV232 f_Veillonellaceae | ASV114 o_Clostridiales |  | ASV104 Mitsuokella |
| ASV86 f_Lachnospiraceae | ASV329 unclassified | **d 90** | ASV310 f_Christensenellaceae |
| ASV164 Roseburia | ASV273 f_Pirellulaceae | ASV2 Streptococcus | ASV377 f_S24-7 |
| ASV83 Prevotella | ASV343 f_[Mogibacteriaceae] | ASV69 unclassified | ASV234 Bifidobacterium |
| ASV191 Oscillospira | ASV342 unclassified | ASV95 f_Succinivibrionaceae | ASV289 f_[Mogibacteriaceae] |
| ASV166 Desulfovibrio |  | ASV107 f_Veillonellaceae | ASV481 o_Bacteroidales |
| ASV135 Coprococcus |  | ASV44 Oscillospira | ASV500 f_Lachnospiraceae |
| ASV182 Oscillospira |  | ASV62 Dorea | ASV183 o_Clostridiales |
| ASV236 Prevotella |  | ASV78 Prevotella | ASV223 Prevotella |
| ASV319 Dialister |  | ASV195 f_Lachnospiraceae | ASV383 Oscillospira |
| ASV170 Ruminococcus |  | ASV131 Coprococcus | ASV282 Treponema |
|  |  | ASV102 Bulleidia | ASV287 o_Bacteroidales |
|  |  | ASV136 Blautia | ASV350 Oscillospira |
|  |  | ASV148 f_Ruminococcaceae |  |
|  |  | ASV163 f_Ruminococcaceae |  |

Fig. S1. Longitudinal dynamics of alpha diversities (chao1, observed_features, and Shannon) of the gut microbiota in recipient pigs gavage fed with PBS with 20% glycerol (Con), fecal microbiota from a nursery pig with low (NL) or high body weight (NH), fecal microbiota from a growing pig with low (GL) or high body weight (GH), and fecal microbiota from a finishing pig with low (FL) or high body weight (FH) at weaning (a). Alpha diversities (chao1, observed_features, and shannon) of the six donors (b).

Fig. S2. Bray-Curtis dissimilarity-based PCOA plots of gut microbial communities from donors and recipient pigs on d 25, 35, 49, 63, and 174. Points represent the gut microbiota in recipient pigs gavage fed with PBS with 20% glycerol (Con/yellow), fecal microbiota from a nursery pig with low (NL/light red) or high body weight (NH/dark red), fecal microbiota from a growing pig with low (GL/light green) or high body weight (GH/dark green), and fecal microbiota from a finishing pig with low (FL/light purple) or high body weight (FH/dark purple) at weaning (a). Donors are yellow color filled triangles (light BW) and squares (heavy BW).

Fig. S3. The top 50 estimated age (EMA)-associated features identified by the regression-based random forest.

Fig. S4. Boxplots present the estimated microbiota ages (EMA) of the gut microbiota in recipient pigs on d 32, 35, 49, 63, and 104 (a). Distributions of EMA values from each group on d 35 and 49 (b). The relative abundances of potential modulated bacterial members associated with EMA on d 35 and 49 by fecal microbial transplantations (c).


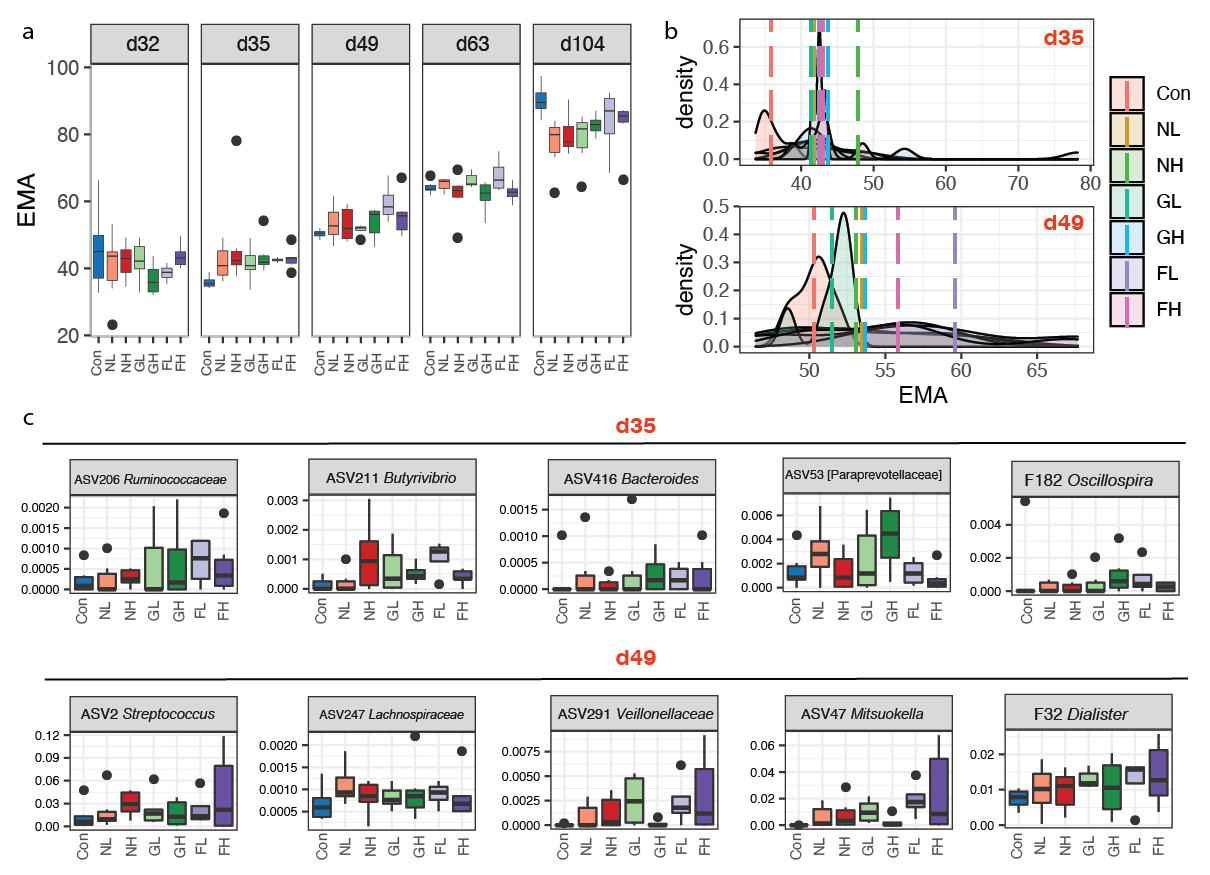


Fig. S5. Boxplots showing the relatives abundances of BW-associated bacterial features at each treatment (a. d 63 and 104; b. d 35; c. d 49). The scatter plots with regression line showing correlations of these features with body weight (BW) on each day. Box color represents different FMT groups (Con/blue: vehicle group; NL/light red: nursery stage microbiota from a light BW donor; NH/dark red: nursery stage microbiota from a heavy BW donor; GL/light green: growing stage microbiota from a light BW donor; GH/dark green: growing stage microbiota from a heavy BW donor; FL/light red: finishing stage microbiota from a light BW donor; FH/dark red: finishing stage microbiota from a heavy BW donor)

Fig. S6. Abundances of the predicted metabolic pathways in the recipient gut microbiotas that were gavage fed either a placebo or growing stage microbiotas from a heavy body weight donor (GH) on d 104.

Fig. S7. Abundances of the influenced metabolic pathways predicted in the recipient gut microbiotas that were gavage fed growing stage microbiotas from both light (GL) and heavy body weight donors (GH) on d 49.

Fig. S8. Abundances of the influenced metabolic pathways predicted in the recipient gut microbiotas that were gavage fed either placebo or growing stage microbiotas from a heavy body weight donor (GH) on d 49.
